# Youth Mental Health in Crisis: Understanding the Relationship Between Mental Health and Physical Pain in Lebanon’s Youth – A Scoping Review

**DOI:** 10.3389/ijph.2025.1608156

**Published:** 2025-04-17

**Authors:** Tanya Tandon, Yara Rouhana, Elias Rahme, Nadine Zalaket, Chantal Martin-Soelch

**Affiliations:** ^1^ Unit of Clinical and Health Psychology, University of Fribourg, Fribourg, Switzerland; ^2^ The Department of Psychology and Social Sciences, Holy Spirit University of Kaslik, Jounieh, Lebanon

**Keywords:** mental health, physical pain, Lebanese youth, depression, anxiety and stress

## Abstract

**Objectives:**

The mental health crisis among young adults in Lebanon, worsened by events like the Beirut Blast and economic instability, requires urgent attention. Globally, 10%–20% of individuals aged 18–29 face mental health challenges, with many also experiencing physical pain. Despite growing evidence of the bidirectional relationship between mental health and pain, this intersection remains underexplored in Lebanon, especially compared to WEIRD countries. This scoping review examines the relationship between physical pain and mental health issues—anxiety, depression, and stress—among Lebanese youth.

**Methods:**

A systematic review of studies from January 2014 to February 2024 was conducted by screening PubMed, PsychInfo, and ScienceDirect. A total of 33 studies were included.

**Results:**

The findings indicate a bidirectional link between mental health and physical pain. University students (36.1% of studies) were particularly impacted, and 81% of studies reported higher pain prevalence among females. Additionally, mindfulness meditation was identified as a potential protective factor, although it remains underexplored in Lebanon.

**Conclusion:**

Addressing these gaps supports tailored interventions for Lebanese youth and enriches our understanding of mental health in non-WEIRD contexts.

## Introduction

Mental health challenges among youth aged 18–29 have become a critical global concern, with 10%–20% of this demographic experiencing significant mental health issues [1]. This concern is particularly acute in Lebanon, where ongoing economic crises, political instability, and the aftermath of traumatic events such as the 2020 Beirut explosion and the COVID-19 pandemic have exacerbated mental health struggles among the youth [2]. Lebanese youth are increasingly affected by severe mental health problems, including stress, anxiety, depression, and post-traumatic stress disorder (PTSD) [3]. The International Labor Organization reports a staggering 23% unemployment rate among Lebanese youth [4], further adding to their financial and psychological burdens. Prior to the current crisis, approximately 25% of young adults in Lebanon experienced high rates of PTSD, with prevalent conditions such as depression (12.6%) and anxiety (16.7%) [5, 6]. Additionally, exposure to frequent violent conflict has left 70% of the population traumatized [7], with a significant proportion of young individuals reporting major depressive disorder, stress-related issues and other anxiety-related conditions [8].

In addition to mental health issues, physical pain has emerged as a major health problem among young populations globally, with around 54% of them reporting physical pain annually [9]. In WEIRD countries (Western, Educated, Industrialized, Rich, and Democratic), common symptoms of physical pain were found to be headaches (8%–83%) and abdominal pain (4%–53%), followed by musculoskeletal pain (4%–40%) and back pain (12%–24%) [10]. According to the Global Burden of Disease, physical pain was the second-highest contributor to global disability in 2018, with 1.9 billion people affected by recurring conditions such as headaches, low back pain, and neck pain—recognized as leading causes of disability [11, 12].

Physical pain was shown to be often associated with mental health issues, such as stress, anxiety and depression [13]. Individuals with depression, stress or anxiety report experiencing physical pain, and the presence of physical pain can also hinder the treatment of mental health conditions [13, 14]. Pereira et al. found that individuals with physical pain were four times more likely to suffer from stress, anxiety or depression than those without pain (Odds ratio [OR] = 4.1) [15]. Conditions such as back pain and stress, anxiety or depression significantly increase the risk of disability, and the co-occurrence of these issues exacerbates this risk [16, 17]. Most of the research on the relationship between mental health and physical pain has been well-documented in the WEIRD cultural contexts where healthcare access, cultural norms, and stressors differ significantly from those in non-WEIRD countries particularly in Middle Eastern regions like Lebanon that are affected by recurrent crises and ongoing wars.

Despite growing recognition of the link between mental health and physical pain, research within the Lebanese youth population remains scarce. Available studies suggest a high prevalence of physical pain conditions such as lower back pain (44.8% in Lebanese workers aged 20–64 [18]) and migraines (35.8% in young adults aged 18–29 [19]). However, these studies do not comprehensively explore the mental health correlates of physical pain in Lebanon’s youth. While evidence from India (non-WEIRD nation) and Switzerland (WEIRD nation) suggests strong links between stress, anxiety, depression, and pain [20], the extent to which these findings apply to Lebanon remains unclear. Given Lebanon’s unique sociopolitical stressors, including economic collapse and exposure to trauma [21], a scoping review is needed to consolidate existing data and identify key gaps. Most existing studies have been conducted in high-income, resource-rich settings, which do not accurately reflect Lebanon’s reality. Therefore, there is a pressing need for research to understand the interconnections between mental health and physical pain within the Lebanese context, particularly among the youth who are bearing the brunt of the country’s crises. This scoping review aims to systematically explore the relationship between physical pain and mental health issues among Lebanese youth. Specifically, it will examine the association between pain and conditions such as anxiety, depression, and stress—links that are well-documented in WEIRD countries but remain underexplored in Lebanon. By synthesizing existing research, this review seeks to provide a comprehensive understanding of how physical pain may be linked to mental health challenges in the Lebanese context. This will not only shed light on an understudied area but also provide valuable insights for future research, interventions, and support services tailored to improve both the physical and mental wellbeing of the youth in Lebanon.

## Methods

### Search Strategy and Inclusion Criteria

This scoping review was conducted in accordance with the PRISMA-ScR (Preferred Reporting Items for Systematic Reviews and Meta-Analyses for Scoping Reviews) guidelines. A systematic literature search was performed across three major databases—PubMed, PsychInfo, and ScienceDirect—to identify studies investigating the relationship between physical pain and mental health issues in Lebanese youth (aged 18 years and above).

#### Search Strategy and Keywords

The search terms included “somatic symptoms,” “chronic pain,” “musculoskeletal pain,” “physical aches,” “somatization/somatization,” “somatic distress,” “mental health related somatic symptoms,” “unexplained medical symptoms,” “Lebanese youth,” “emergent youth/adult,” “adults in Lebanon,” “Lebanese adults,” “mental health disorders,” “anxiety,” “depression,” “stress,” “Lebanese youth,” and “non-WEIRD populations.” The search spanned January 2014 to February 2024 and included peer-reviewed studies written in English. Grey literature, conference abstracts, and case studies were excluded to maintain methodological rigor.

#### Inclusion and Exclusion Criteria

Studies were included if they i) studies that showed the relationship between physical pain and mental health ii) focused on Lebanese adults only (over 18 years of age), iii) were published in English, and iv) were published between January 2014 – February 2024. Studies were excluded if they: i) focused on Syrian refugees in Lebanon and ii) did not assess the interaction between mental health and physical pain. *Rationale for Exclusion of Syrian Refugees:* Syrian refugees in Lebanon face unique socio-economic conditions, mental health challenges, and barriers to higher education that differ significantly from the broader Lebanese young adult population. Their experiences of displacement, trauma, and limited healthcare access introduce distinct stressors that fall outside the scope of this review. To ensure a focused analysis on Lebanese young adults, studies on refugee populations were excluded.

#### Study Selection and Data Extraction

After screening 737 studies, 33 met inclusion criteria. The lead author (TT) conducted the initial screening, with a second reviewer (YR) verifying selected studies. Discrepancies were resolved through consensus. Data extracted included study design, sample size, pain type, mental health outcomes, and key findings.

### Data Analysis

**FIGURE 1:**
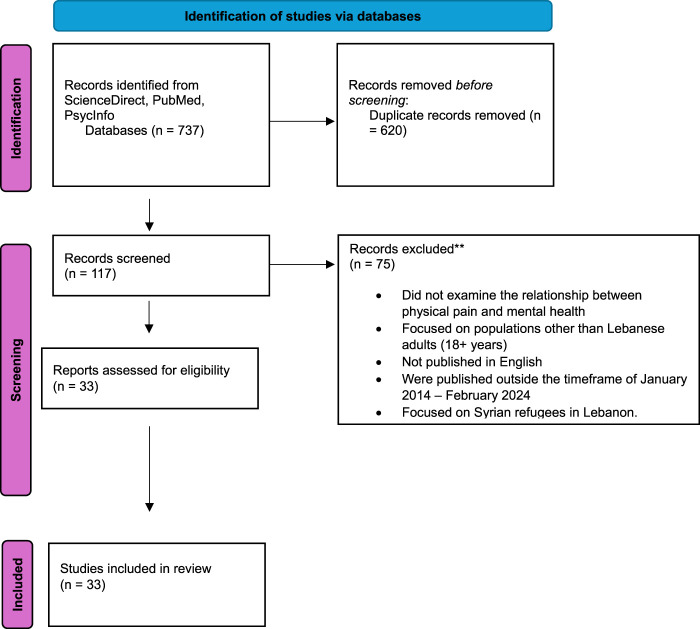
PRISMA flow diagram of study selection (Lebanon, 2025).

After screening and checking the articles for eligibility based on hand-searched, we found a total of 33 articles that were exported to excel. This was carried out by the lead author (TT), with decisions reviewed by second author (YR). Where discrepancies in classification existed, the article in question was discussed and agreement reached between authors. We present our results in the PRISMA flow chart (Figure 1).

## Results

### Search Results

A total of 33 studies were included in this review, summarizing the relationship between physical pain and mental health problems. The study selection process is depicted in Figure 1, and the characteristics of the studies are summarized in Table 1. Among these, 13 studies (36%) were conducted on university students between 18 and 26 years of age [22, 25, 28, 30, 32, 33, 38, 40–42, 44, 52, 54] while the remaining 23 studies (63.8%) examined individuals diagnosed with chronic pain and healthcare professionals, such as nurses [23, 24, 26, 27, 29, 31, 34–37, 39, 43, 44, 46, 48–51, 53, 55–57]. Of the included studies, 29 (81%) had majority of females participants [22, 23, 25–32, 34–37, 39–42, 44–47, 52–57] and in 30 studies (83.3%), quantitative self-report measures were the most common method of assessing physical pain and mental health [24, 26–30, 32, 34, 35, 37–49, 51, 53–57].

**TABLE 1:** Summary of extracted studies and their characteristics (Lebanon, 2025).

Author(s), Year	Study Design	Sample Size (N)	Population	Setting	Pain Assessment	Measures of Mental Health	Key Findings
Beydoun and Mehling [22]	Observational study	554	Lebanese residents (18–64 years, 78.7% between 18 and 28 years, majority female)	Online Survey	Interoceptive awareness (MAIA-2)	Traumatic events (THS), traumatic experiences integrating in one’s identity (CES), PTSD symptoms (PCL-5)	Trauma centrality and interoceptive awareness significantly predict PTSD severity, even after accounting for trauma type or frequency, with Lebanese populations showing heightened PTSD risk and trauma manifesting physically (e.g., fatigue, pain).
Jahrami et al. [23]	Observational Study (Matched case-control study)	120	Patients with depression, aged between 20 and 60 years, 59% were females.	Clinical setting	Questionnaire included demographics, medical history, Physical activity (IPAQ-SF)	Psychiatric history for cases, smoking status, and dietary intake (FFQ)	Patients with depression consumed more calories and macronutrients, smoked three times more, and exercised less. They faced twice the risk of obesity, type 2 diabetes, hypertension, and musculoskeletal disorders.
Jaoude et al. [24]	Epidemiological Study	314	Dentists, (Age 39.2 ±11.66, 184 male and 130 female)	Interview	Musculoskeletal pain asked using interview	Interview	High prevalence of musculoskeletal disorders (61.5%) among Lebanese dentists, with women more affected than men. Lack of a dental assistant increased neck pain, while sports participation was linked to a lower risk of tendinitis.
Akiki et al. [25]	Cross-sectional study	528	Lebanese adults (Mean age: 24.51 ± 7.68, 65.6% female)	Online survey	Computer Vision Syndrome (CVSS), Migraine (MIDAS)	Insomnia (LIS-18), Stress (BDS-10)	Stress mediated CVS, migraine, and insomnia, insomnia linked to stressful life events.
Barake et al. [26]	Cross-sectional study	300	Pharmacists (Mean age 30.9 ± 9.1, 62.3% female)	Clinical setting, Lebanon	Chronic diseases and symptoms (Chronic disease self-management program questionnaire)	Behavioral and Psychosocial Patterns: Smoking habits,alcohol, physical activity, stress, work satisfaction (Chronic disease self-management program questionnaire)	Despite a lower prevalence of chronic diseases, community pharmacists face significant challenges related to physical pain, unhealthy lifestyle behaviors and mental health symptoms.
Bou Ali et al. [27]	Cross-sectional study	1292	Lebanese participants over 40 years, more females	Clinical setting	Stroke symptoms (QVSFS), Physical Pain symptoms (SF-12)	Quality of Life (SF-12)	Arthritis (knee pain) prevalence is higher among those with symptoms and a history of stroke and leading to decrease in quality of life.
Chahine et al. [28]	Observational, descriptive, monocentric, cross-sectional study	471	University students (18–28 years, 58.4% female, 41.6% male)	Online survey	Headache assessment (IHS; MIDAS)	Insomnia (LIS-18), Anxiety (LAS), Depression (PHQ-9)	High prevalence of migraine linked to insomnia, stress, anxiety, depression and it is more common in females.
Chalhoub et al. [29]	Cross-sectional study	1840	Lebanese adults, (18+, mean 26.6 ±8.8, 62% female)	Online survey	Physical pain measured through interview at the Baseline	Depression (PHQ9), Anxiety(GAD-7), Fear of COVID-19 (FCV-19S), Death Anxiety (ASDA)	Physical pain is linked to fear of death due to COVID-19, anxiety, depression, and sleep disturbances.
Costanian et al. [30]	Cross-sectional study	813	University students (18 years, 325 male, 488 female)	Clinical setting	IBS: Rome III criteria	Psychological Stress through patient interview	IBS exacerbated by lack of exercise and psychological factors related to stress.
Dahham et al. [31]	Cross-sectional retrospective health-related quality of life (HRQoL) study	210	Patients with multiple sclerosis (MS) (mean age: 43.3 years, 65.7 % female)	Clinical setting	Pain/discomfort: The health dimension of (EQ-5D-5 L)	Patients’ mobility, self-care, usual activities, Anxiety/Depression (EQ-5D-5), Overall perception of health (EQ-VAS), Symptoms and psychological well-being (MusiQoL)	People with musculoskeletal pain in Lebanon experience declining quality of life as the pain progresses, with mobility, pain, and mental health issues being prevalent; factors like higher education, employment, and lower disability scores correlate with better quality of life.
El Hasbani et al. [32]	Cross-sectional study	2178	University students (18 and 25 years, mostly female)	Online survey	Pain (WPI; SSS)	Anxiety, depression, social dysfunction (GHQ-12)	Fibromyalgia prevalent in students, more common in females, associated with anxiety, depression, and social dysfunction
Fares et al. [33]	Clinical observation	93	Lebanese adults (16–26 years, 87 men, 6 women)	Clinical setting	Pain Localization: Self-identified pain locations	Psychological and social factors: patient interviews, parental input	LBP is linked to mental health issues like depression, other psychological symptoms in 19%
Gerges et al. [34]	Cross-sectional study	452	Patients (mean age: 47.60, 52.7% female)	Clinical setting	Pain (Survey with list of 38 stressors)	Stressors during hospitalization (Survey with list of 38 stressors), depression(PHQ-9), anxiety (LAS-10), perceived social support (MSPSS) and spiritual well-being (FACIT-Sp-12)	Hospitalized patients report high levels of pain which is linked to higher anxiety and depression and leading to illness apprehension, hopelessness, and financial burden. Spiritual well-being and social support mitigate these effects
Ghoussoub et al. [35]	Cross-sectional retrospective study	537	Healthcare workers (Mean age 34, 75% female)	Clinical setting	Anthropometric data & questions about back pain duration, trigger, risk factors, history through an interview	Interview	LBP is highly prevalent, especially in females. Primary prevention via posture education and proper equipment is crucial
Godah et al. [36]	Case-control study	260	Lebanese adults (>15 years old, 60% female)	Work place	Pain: shoulder pain, tenderness, or stiffness (visual analog scale ≥6)	Behavioral and Psychosocial Patterns: Smoking habits,alcohol, physical activity, stress were asked through an interview	Chronic shoulder pain associated with high stress, depression, job dissatisfaction, and poor quality of life
Hajj et al. [37]	Prospective Study	143	Participants (Mean age 34.32 ± 11.88, 81 % female)	Clinical setting	Migraine (ICHD), IBS (Rome III criteria)	Anxiety, stress and Potential triggering factors (dietary habits, hormonal, psychological/emotional, environmental factors (questionnaire not specified)	Emotional factors, such as anxiety, stress, and annoyance, are the most common triggering factors for both IBS and migraines. Patients having migraine and IBS together are more likely to experience a concomitant chronic disease or a psychiatric disorder
Hatem et al. [38]	Observational cross-sectional study	401	University medical students (18–30 years, 45.9% male and 54.1% female)	Online survey	Physical Pain symptoms (SF-12)	Quality of Life (SF-12)	The COVID-19 pandemic significantly impacted medical students' mental health and quality of life, with those attending online sessions reporting higher bodily pain and mental health scores but lower emotional role scores
Huijer et al. [39]	Cross-sectional, psychometric validation study	Study 1(150), Study 2(44)	Study 1: Patients with chronic nonmalignant pain (Mean age 49.03, 67% female); Study 2: University students	Clinical setting and Educational setting	Pain Catastrophizing (PCS-A), Physical pain symptoms (BPI; EORTC QLQ-C30, physical subscale)	Cognitive, emotional, and social factors (EORTC QLQ-C30; GHS), Depression (CES-D—Arabic Version)	Pain catastrophizing is strongly linked to increased depression, higher pain intensity, reduced quality of life, and heightened pain sensitivity, even when accounting for various demographic and clinical factors
Ismail et al. [40]	Cross-sectional study	422	Lebanese adults (Mean age 25.08, 60% female)	Online survey	Somatic symptoms (A-DSSS)	Depression (PHQ-9 & A-DSSS), Alcohol use (AUDIT-C), Smoking History	Gender, physical activity, alcohol and smoking linked to somatic symptoms and depression
Kabbara et al. [41]	Cross-sectional study	502	Lebanese university students, (18 − 25-year-old)	Online & Educational setting	Anthropometric and menstrual cycle characteristics (Questionnaire)	Lifestyle behaviors: smoking habits, physical activity, adherence to low-calorie or vege- tarian diets, and degree of daily stress (Questionnaire)	Menstrual pain is prevalent in Lebanese university students. Stress contributes to the discomfort, while vegetarian or low-calorie diets act as a protective factor
Kawtharani et al. [42]	Literature review	342	Dentists (18-35 years, 41.2% female)	Review	Musculoskeletal pain symptoms (EAQ; REBAS; NDI)	Questionnaires (not speficied) related to stress and anxiety	No correlation between work hours and pain, psychological factors like stress and anxiety linked to neck pain that affects daily tasks
Khazaal et al. [43]	Prospective observational epidemiological study	143	Stroke survivors (mean age, 72.46 years; 60.7% male)	Clinical setting	Neuropathic pain (DN4)	Cognitive function(MMSE), depression & anxiety (HADS), fatigue (FSS), neurological function (NIHSS)	Post-stroke survivors face high rates of fatigue, cognitive impairment, depression, anxiety, and neuropathic pain. Depression is linked to anxiety, pain, and physical activity
Najem et al. [44]	Double-blind randomized controlled experiment	208	University students (18–25 years, 82% female)	Clinical setting	Physical Pain (PPT; CPM; NPRS)	Style of praying (PFS)	Prayer as mindfulness based meditation positively affects pain sensitivity and intensity, as a protective factor
Nehme et al. [45]	Cross-sectional study	403	Adults (Mean age 32.76 ± 13.24, 65% female)	Online Survey	Physical symptoms of pain (PHQ-15)	Depression (PHQ-9),stress (BDS-10), PTSD (PCL-C), anxiety (LAS-10)	PTSD, COVID pandemic, anxiety, stress and depression were significantly associated with more somatization, COVID vaccine associated with less somatization
Nehme et al. [46]	Cross-sectional study	264	Lebanese residents (mean age of 32.76 ± 13.24, female)	Online survey	Physical symptoms of pain (PHQ-15)	Depression (PHQ-9), Stress (BDS-10), PTSD (PCL-C), Anxiety (LAS-10), Financial distress & wellbeing(IFDFW)	A worse financial wellbeing was significantly associated with more depression, which was associated with more somatization. A worse financial wellbeing was significantly and directly associated with more somatization
Nehme et al. [47]	Cross-sectional study	403	Adults (Mean age 32.76 ± 13.24, 66% female)	Online Survey	Physical symptoms of pain (PHQ-15)	PTSD symptoms (PCL-C), Emotion regulation (ERQ)	Higher PTSD symptoms, particularly from the COVID-19 pandemic, are strongly linked to increased somatization, with expressive suppression moderating this relationship
Saleh et al. [48]	Cross-sectional study	81	Patients with Spinal Cord Injury (SCI) (mean age 38.15 ± 11.75, majority male)	Online Survey	Physical pain symptoms (BPI; DN4; SF-12)	Quality of Life (SF-12)	High prevalence of chronic pain (mostly neuropathic). Employment status, pain type, and severity affected pain interference and quality of life
Saleh et al. [49]	Cross-sectional study	132	Patients with chronic non-specific low back pain (CNSLBP) (18–35 years, 70% male)	Online Survey	LBP intensity (NRS), Bodily pain (PCS)	Vitality, emotional role, social functioning, and mental health (MCS) and quality of life (SF-12)	Significant correlation found between pain intensity, PCS, and MCS. Less educated participants were less likely to perceive physical therapy for pain
Summaka et al. [50]	Cross-sectional study	118	Participants with physical disabilities (Mean age 37.75 ± 11.33, 88.1% male)	Online survey + Interview	Interview	Psychoneurotic complaints, depression and anxiety (HSCL-25), fear from COVID-19 (A-FCV–19S)	A significant portion of individuals with physical disabilities experienced mild fear of COVID-19, anxiety, and depression. Factors like age, education, and employment status were associated with these conditions
Sunna et al. [51]	Case control study	102	Patients with spinal cord injury	Clinical setting	Physical health (SF-36)	Quality of life (SF-36), depression (PHQ-9-A), anxiety(GAD-7)	Spinal cord injury patients experience reduced quality of life, with depression, anxiety, and pain being significant contributors
Tahhan et al. [52]	Cross-sectional study	1144	University students (18–25 years, majority female)	Educational setting	Migraine headaches (three-item ID Migraine™ screener)	Behavioral Patterns: Smoking habits,alcohol consumption, eating habits, smoking, sleeping	University students experience a high prevalence of migraines, with triggers varying between public and private institutions. Public university students often report fasting as a trigger, possibly due to financial constraints or academic pressures, while private university students are more susceptible to smoking-induced headaches, which may be linked to stress and lifestyle factors
Younan et al. [53]	Cross-sectional study	2,852	Nurses, (Age 30+, 79% female)	Healthcare setting	Work-related musculoskeletal disorders (6 investigator-developed items)	Chronic occupational fatigue (OFER)	High correlation between musculoskeletal disorders, chronic fatigue and physical pain, stress, and work factors
Younes et al. [54]	Cross-sectional study	318	Lebanese university female students (18+)	Online survey	Premenstrual Symptoms (PSST)	Stress (Holmes-Rahe Life Stress inventory), depression (LDS-19), Childhood Trauma(CASRS)	Stressful life events and abuse correlated with higher depression and Pre-menstrual pain symptoms

Notes: A-DSSS: Arabic version of Depression and Somatic Symptoms Scale; A-FCV-19S: Arabic Fear of COVID-19 Scale; ASDA: Arabic Scale of Death Anxiety; AUDIT-C: Alcohol Use Disorder Identification Test; BDS-10: Beirut Distress Scale-10; BPI: Brief Pain Inventory; CASRS: Child Abuse Self-Report Scale; CES: Centrality of Events Scale; CES-D—Arabic Version: Arabic Center for Epidemiological Studies Depression Scale; CPM: Conditioned Pain Modulation; CVSS: Computer Vision Syndrome Scale; DN4: Douleur Neuropathique en 4; DSM-5: Diagnostic and Statistical Manual of Mental Disorders, Fifth Edition Criteria; EAQ: Ergonomic Awareness Questionnaire; EORTC QLQ-C30: European Organization for the Research and Treatment of Cancer Quality of Life Questionnaire-30; EQol: European Quality of life; EQ-VAS: EuroQol-Visual Analogue Scales; ERQ: Emotion Regulation Questionnaire; FACIT-p-12: Functional Assessment of Chronic Illness Therapy - Spiritual Well-Being 12; FCV-19S: Fear of COVID-19 Scale; FFQ: Food Frequency Questionnaire; FSS: Fatigue Severity Scale; GAD-7: Generalized Anxiety Disorder-7; GHQ-12: General Health Questionnaire-12; GHS: Global Health Scale; GPES: Global Perceived Effect Scale; HADS: Hospital Anxiety and Depression Scale; HSCL-25: Hopkins Symptom Checklist-25; IBS: Irritable Bowel Syndrome; ICHD: International Classification of Headache Disorders; IHS: International Headache Society; IFDFW: InCharge Financial Distress/Financial Well-Being Scale; IPAQ-SF: International Physical Activity Questionnaire-Short Form; LAS: Lebanese Anxiety Scale; LDS-19: Lebanese Depression Scale-19; LIS-18: Lebanese Insomnia Scale-18; LBP: Lower Back Pain; MAIA-2: Multidimensional Assessment of Interoceptive Awareness Version 2; MIDAS: Migraine Disability Assessment Score; MMSE: Mini-Mental State Examination; MusiQoL: Multiple Sclerosis International Quality of Life Questionnaire; MCS: Mental Component Summary; NDI: Neck Disability Index; NIHSS: National Institutes of Health Stroke Scale; NPRS: Numerical Pain Rating Scale; NRS: Numeric Rating Scale; OFER: Occupational Fatigue Exhaustion Recovery; PCL-C: PTSD Checklist–Civilian Version; PCS-A: Pain Catastrophizing Scale-Arabic Version; PFS: Prayer Function Scale; PHQ-9: Patient Health Questionnaire-9; PHQ-15: Patient Health Questionnaire-15; PPT: Pressure Pain Threshold; PSST: Premenstrual Symptoms Screening Tool; PCS: Physical Component Summary; QVSFS: Questionnaire for Verifying Stroke-Free Status; REBAS: Rapid Entire Body Assessment Scale; SF-12: Short Form Health Survey-12; SSS: Symptoms Severity Score; THS: Traumatic History Screen; WPI: Widespread Pain Index.

Only six studies adopted a qualitative approach using open-ended questionnaires to evaluate physical pain [23, 31, 33, 36, 50, 52].

### Types of Physical Pain and the Tools Used to Assess

The review identified various types of physical pain frequently reported among participants, particularly university students and females. The most common types of pain included: musculoskeletal pain [24, 53], neck and lower back pain [35, 42, 48], migraine [25, 28, 52], and menstrual pain [41, 54]. These findings suggest that physical pain is highly prevalent among university students, with a particularly high burden among female participants [28, 30, 32, 38, 40, 52]. In these studies, the physical pain was measured using Premenstrual Symptoms Screening Tool (PSST) [54], Depression and Somatic Symptoms Scale (A-DSSS) [40], Pain Catastrophizing Scale-Arabic Version (PCS-A) [39], Patient Health Questionnaire (PHQ-9) [46], Neck Disability Index (NDI), Numeric rating scale (NRS) [42, 44, 48] Arabic version of Brief Pain Inventory (BPI-A) [39, 48], Beirut Distress Scale (BDS-10) [25], Multidimensional Assessment of Interoceptive Awareness Version 2 (MAIA-2) [22], Short Form Health Survey (SF-36) [27, 38], Neuropathic Pain Questionnaire (DN4) [39, 48], Migraine disability assessment test (MIDAS) [25, 28], standardized self-reported questionnaire to measure Irritable Bowel Syndrome [30], Widespread pain index (WPI) and Symptoms severity score (SSS) [32].

### Mental Health Problems Associated With Physical Pain

A strong association between physical pain and mental health problems was observed across all included studies. The most frequently reported mental health concerns were stress, depression and anxiety that were strongly related to physical pain [25, 28, 30–34, 36, 42, 53]. In some of the studies, they acted as a predictor [25, 42, 53, 54], while in others they were the consequence of physical pain [31, 32, 34, 41]. This is in line with many studies conducted on pain [58–62]. Interestingly, only one study reported mindfulness meditation as a protective factor against the development of pain [44].

### Gender Differences in Pain and Mental Health

Gender differences were evident in both the prevalence and intensity of physical pain and mental health symptoms. Female participants reported significantly higher levels of pain, particularly menstrual pain and migraines, which were associated with increased depressive symptoms [28, 32, 40].

### Assessment Methods Used: Gaps and Limitations

Most studies reviewed relied heavily on quantitative measures [37, 38, 45, 46, 54], to assess pain and other mental health problems, with only 2 studies employing a mixed-methods approach [23, 50] that combined both qualitative and quantitative data. Additionally, only a few studies utilized the well-validated Arabic version of the scales like A-DSSS) [40], PCS-A [39], BDS-10 [25], and BPI-A [39, 48], emphasizing a significant gap in the cultural validation of assessment tools.

## Discussion

This scoping review addresses a significant research gap by examining the relationship between physical pain and mental health issues among young people in Lebanon—a non-WEIRD nation affected by ongoing conflict and recurring crises. By focusing on Lebanon, our study aims to deepen understanding of physical pain and its mental health associations within diverse cultural settings, ultimately contributing to a more inclusive global perspective on the relationship between physical and mental health in underrepresented populations.

One major finding from this review is the strong bidirectional relationship between various types of physical pain, particularly among university students in Lebanon, and mental health issues like depression, anxiety, and stress [25,42]. This pattern aligns with previous research conducted in Switzerland [63] and India [64] which also showed similar relationships among university students. In some studies, mental health issues such as stress, depression, and anxiety were identified as predictors of physical pain [25, 42, 53, 54] while in others, they appeared to result from physical pain [31, 32, 34, 41]. These findings resonate with broader research indicating that chronic pain and mental health often interact in a cyclical way, where pain worsens mental health issues, and *vice versa* [58–62].

Approximately 36% of the studies included in this review focused specifically on university students aged 18–26 [22, 28, 30]. Among these students, the most common forms of pain reported were musculoskeletal pain [24, 53], neck and lower back pain [35, 42, 48], migraine [28, 52], and menstrual pain [41, 54]. The prevalence of these types of pain highlights unique vulnerabilities within this demographic, potentially stemming from academic stress, lifestyle factors, or societal expectations [65, 66]. The remaining 64% of studies [5, 23, 44] involved individuals with chronic pain and healthcare professionals, like nurses, who might experience high levels of work-related stress and physical strain [67, 68].

A notable finding from these studies is the demographic focus, as 81% of studies had a majority of female participants [32, 36, 37]. This gender disparity in the experience and reporting of pain is consistent with previous literature, which often suggests that women tend to report higher levels of both acute and chronic pain [69–71]. Various factors may contribute to this trend, including biological differences, such as hormonal fluctuations that can influence pain sensitivity [72, 73], as well as psychosocial factors, such as differing gender norms related to expressing pain [74, 75]. In many cultures, including Lebanon, women may feel more socially permitted to express pain and seek help [76], while men may face societal expectations to endure pain quietly due to norms around masculinity [77]. Additionally, research suggests that women are more likely to experience somatic symptoms associated with mental health conditions, such as depression and anxiety, which could further explain the higher reported prevalence of physical pain among females [78–81]. These findings underscore the importance of considering gender-specific factors when assessing and treating physical pain and mental health issues among young people, as this can lead to more targeted and effective interventions.

Furthermore, the 83.3% of the studies predominantly used quantitative self-report measures to assess physical pain and mental health [24,40,41]. The measurement tools to assess and mental health problems included scales such as the PSST [54], A-DSSS [40], BDS-10 [25], PCS-A [39], PHQ-9 [47], NRS [42, 44, 48], BPI-A [39, 48], MAIA-2 [22], SF-36 [27, 38], DN4 [39, 48], MIDAS [25, 28], WPI [32] and SSS [32]. These tools provide standardized metrics for assessing pain levels and mental health symptoms, although the review points out a lack of culturally validated tools for use in Lebanese populations. Only a few studies used the culturally validated versions of established tools like the A-DSSS, BDS-10, PCS-A, and BPI-A [25, 39, 40, 48], emphasizing a significant gap in culturally appropriate assessment methods. In addition, these quantitative measures, may not fully capture the culturally specific experiences of pain and mental health [82]. Pain can be expressed differently across cultures [83, 84] and relying solely on quantitative methods or tools designed for WEIRD populations risks overlooking culturally nuanced experiences of pain and mental health issues [85]. In the context of Lebanon, a non-WEIRD population, there is a crucial need for culturally adapted instruments. Moreover, incorporating qualitative methods would provide a more in-depth understanding of how pain is experienced and expressed within this cultural context [86], potentially leading to more accurate and relevant assessments. This gap underscores the importance of expanding research methodologies to ensure the tools used are both culturally sensitive and inclusive, particularly when addressing mental health and pain in diverse populations.

An intriguing finding was the emergence of mindfulness meditation as a potential protective factor, identified in only one study [44]. Despite extensive literature supporting mindfulness as effective in reducing chronic pain and improving mental health outcomes [87–89] this approach appears underexplored in the Lebanese context. This suggests that mindfulness could represent an untapped resource for managing comorbid physical and mental health conditions among Lebanese youth, warranting further investigation.

In summary, this scoping review highlights a substantial association between physical pain and mental health problems among young adults in Lebanon. It also reveals limitations in current methodologies and stresses the need for culturally validated tools. By adopting a more holistic, gender-sensitive, and culturally inclusive approach, future research can better capture the complex experiences of physical pain and mental health in Lebanon, ultimately leading to more effective and targeted mental health strategies for these populations.

### Limitations

Despite its contributions, this scoping review merits limitations. First, most studies focused on quantitative measures, limiting the depth of understanding regarding the lived experiences of pain among youth in Lebanon. The lack of qualitative research also hampers the ability to explore culturally specific expressions of pain. Second, while this review highlights gender differences in the experience of pain, it does not account for other important factors, such as socioeconomic status, that could influence these experiences. Third, due to indexing limitations, studies examining associations between mental health and physical pain that did not include specific keywords in their titles or abstracts may have been overlooked. Additionally, the search strategy was not formally validated or reviewed by a librarian. However, the search terms were carefully developed based on relevant keywords and were guided by the specific aims of the review. Furthermore, we acknowledge that our inclusion criteria did not explicitly differentiate between clinical and community studies or consider grey literature and unpublished studies. This decision was made with the primary aim of understanding the relationship between physical pain and mental health within the Lebanese context, rather than focusing on study setting or type, and to maintain rigor and reliability in the data. Future reviews could address this limitation by expanding the search strategy to include a broader range of pain-related measures or by incorporating grey literature to capture a wider array of relevant studies. Fourth, we did not conduct a formal calibration exercise for data abstraction questions, nor did we calculate the kappa agreement between the two reviewers. While both reviewers independently screened the studies, any disagreements were resolved through discussion and consensus. The absence of a formal kappa calculation may limit the ability to quantify the level of agreement between reviewers. In future reviews, we would like to implement a calibration process and calculate Cohen’s kappa to ensure greater consistency and reliability in the data abstraction process.
